# Prognostic Significance of Pathologic Lymph Node Invasion in Metastatic Renal Cell Carcinoma in the Immunotherapy Era

**DOI:** 10.1245/s10434-023-14367-6

**Published:** 2023-10-10

**Authors:** Lukas Scheipner, Francesco Barletta, Cristina Cano Garcia, Reha-Baris Incesu, Simone Morra, Andrea Baudo, Anis Assad, Zhe Tian, Fred Saad, Shahrokh F. Shariat, Alberto Briganti, Felix K. H. Chun, Derya Tilki, Nicola Longo, Luca Carmignani, Martin Pichler, Sascha Ahyai, Pierre I. Karakiewicz

**Affiliations:** 1 Cancer Prognostics and Health Outcomes Unit, Division of Urology, University of Montréal Health Center, Montréal, QC Canada; 2 Department of Urology, Medical University of Graz, Graz, Austria; 3 Unit of Urology/Division of Oncology, Gianfranco Soldera Prostate Cancer Lab, IRCCS San Raffaele Scientific Institute, Milan, Italy; 4 Vita-Salute San Raffaele University, Milan, Italy; 5 Department of Urology, University Hospital Frankfurt, Goethe University Frankfurt am Main, Frankfurt am Main, Germany; 6 Martini-Klinik Prostate Cancer Center, University Hospital Hamburg-Eppendorf, Hamburg, Germany; 7 Department of Neurosciences, Science of Reproduction and Odontostomatology, University of Naples Federico II, Naples, Italy; 8 Department of Urology, IRCCS Policlinico San Donato, Milan, Italy; 9 Department of Urology, Comprehensive Cancer Center, Medical University of Vienna, Vienna, Austria; 10 Department of Urology, Weill Cornell Medical College, New York, NY USA; 11 Department of Urology, University of Texas Southwestern, Dallas, TX USA; 12 Hourani Center for Applied Scientific Research, Al-Ahliyya Amman University, Amman, Jordan; 13 Department of Urology, University Hospital Hamburg-Eppendorf, Hamburg, Germany; 14 Department of Urology, Koc University Hospital, Istanbul, Turkey; 15 Department of Urology, IRCCS Ospedale Galeazzi–Sant’Ambrogio, Milan, Italy; 16 Division of Oncology, Department of Internal Medicine, Medical University of Graz, Graz, Austria; 17 Department of Hematology and Oncology, Medical Faculty, University of Augsburg, Augsburg, Germany

**Keywords:** mRCC, Lymph node invasion, SEER, Population-based, CSM

## Abstract

**Background:**

This study aimed to test the prognostic significance of pathologically confirmed lymph node invasion in metastatic renal cell carcinoma (mRCC) patients in this immunotherapy era.

**Methods:**

Surgically treated mRCC patients were identified in the Surveillance, Epidemiology, and End Results (SEER) database between 2010 and 2018. Kaplan-Meier plots and multivariable Cox-regression models were fitted to test for differences in cancer-specific mortality (CSM) and overall mortality (OM) according to N stage (pN0 vs pN1 vs. pNx). Subgroup analyses addressing pN1 patients tested for CSM and OM differences according to postoperative systemic therapy status.

**Results:**

Overall, 3149 surgically treated mRCC patients were identified. Of these patients, 443 (14%) were labeled as pN1, 812 (26%) as pN0, and 1894 (60%) as pNx. In Kaplan-Meier analyses, the median CSM-free survival was 15 months for pN1 versus 40 months for pN0 versus 35 months for pNx (*P* < 0.001). In multivariable Cox regression analyses, pN1 independently predicted higher CSM (hazard ratio [HR], 1.88; *P* < 0.01) and OM (HR, 1.95; *P* < 0.01) relative to pN0. In sensitivity analyses addressing pN1 patients, postoperative systemic therapy use independently predicted lower CSM (HR, 0.73; *P* < 0.01) and OM (HR, 0.71; *P* < 0.01).

**Conclusion:**

Pathologically confirmed lymph node invasion independently predicted higher CSM and OM for surgically treated mRCC patients. For pN1 mRCC patients, use of postoperative systemic therapy was associated with lower CSM and OM. Consequently, N stage should be considered for individual patient counseling and clinical decision-making.

**Graphical abstract:**

Consort diagram of the study population.
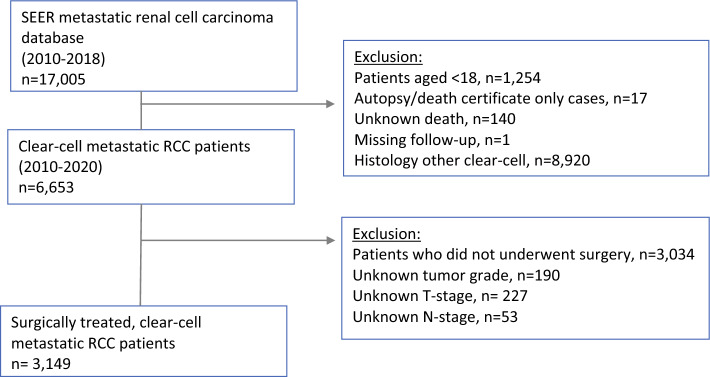

**Supplementary Information:**

The online version contains supplementary material available at 10.1245/s10434-023-14367-6.

Currently, the role of lymph node dissection (LND) in cytoreductive nephrotomy (CN) is controversial and not generally endorsed.^1^ However, historic studies demonstrated that information about lymph node invasion (LNI) status represents an independent predictor of cancer-specific mortality (CSM).^2,3^ Specifically, patients with LNI historically exhibited markedly worse survival than those without LNI (pN1 vs pN0). Based on the absence of contemporary data addressing the potentially added value of confirmed pN1 versus pN0 status at cytoreductive nephrectomy, we addressed this knowledge gap. Specifically, we investigated the prognostic significance of pathologic lymph node involvement in a contemporary cohort of surgically treated metastatic renal cell carcinoma (mRCC) patients relying on the Surveillance, Epidemiology, and End Results (SEER) database.

## Methods

### Data Source and Study Population

In the SEER database (2010–2018), we identified patients 18 years old or older with histologically confirmed unilateral metastatic RCC (International Classification of Disease for Oncology [ICD-O] site code C64.9) who harbored clear-cell histology (ICD-O-3 code 8310). Cases identified only at autopsy were excluded. The study included only mRCC patients who received cytoreductive nephrectomy. Further exclusion criteria ruled out patients with unknown T stage, unknown grade, or missing data on pathologic lymph node status. Autopsy or death certificate-only cases also were excluded (consort diagram).

### Variables of Interest

The demographic covariates consisted of age at diagnosis (years, continuously coded), sex, and race/ethnicity (Caucasians vs others). The tumor characteristics consisted of T stage (T1 vs. T2 vs. T3 vs. T4), grade (G1–G2 vs. G3 vs. G4), and pathologic N stage (pN0 vs. pN1 vs. pNx). Patients who did not undergo lymph node dissection (LND) were labeled pNx.

Systemic therapy comprised only postoperative therapy after cytoreductive nephrectomy and was coded as received or not received. Cancer-specific mortality (CSM) and overall mortality (OM) were the primary end points of the study.

### Statistical Analyses

First, the baseline characteristics of the cohort were analyzed. Descriptive statistics included frequencies and proportions for categorical variables. Medians and interquartile ranges (IQRs) were reported for continuously coded variables.

Second, Kaplan-Meier plots were used to display rates of CSM and OM according to pN0 versus pN1 versus pNx. The association between pathologic N stage (pN0 vs pN1 vs pNx) and CSM as well as OM was further tested in multivariable Cox regression models. Adjustment variables consisted of age, year of diagnosis, sex, T stage, grade, ethnicity, and systemic therapy exposure status.

Third, we reapplied the previously described methodology in a subgroup analysis. Specifically, we analyzed CSM and OM hazard ratios (HRs) of pN1 mRCC patients according to systemic therapy exposure. In all statistical analyses, R software environment for statistical computing and graphics (R version 4.1.2; R Foundation for Statistical Computing, Vienna, Austria) was used. All tests were two-sided, with the level of significance set at a *P* value lower than 0.05.^4^

## Results

### Descriptive Characteristics

Between 2010 and 2018, we identified 3149 cytoreductive nephrectomy mRCC patients (Table 1). Of these patients, 1255 (40%) underwent LND and 1894 (60%) did not (pNx). In terms of disease stage, 443 patients (14%) were labeled as pN1, 812 (26%) as pN0, and 1894 (60%) as pNx. Overall, 29% of all the patients were female, and 13% were not Caucasian. Statistically, the ages of the groups differed significantly (median ages: 62 years for pN1 vs 60 years for pN0 vs 63 years for pNx; *P* < 0.001). Additionally, the pN1 patients exhibited higher rates of T3 tumors (70% for pN1 vs. 69% for pN0 vs. 59% for pNx) and T4 tumors (22% for pN1 vs. 18% for pN0 vs. 14% for pNx) as well as higher rates of G4 tumors (58% for pN1 vs. 39% for pN0 vs. 33% for pNx).

**Table 1:** Descriptive characteristics of 3149 metastatic renal cell carcinoma (mRCC) patients in the Surveillance, Epidemiology, and End Results (SEER) database (2010–2018)

Characteristic	*n*	Overall (*n* = 3149) *n* (%)	pN1 (*n* = 443, 14%) *n* (%)	pN0 (*n* = 812, 26%) *n* (%)	pNx (*n* = 1894, 60%) *n* (%)	*P* value^a^
Median age: years (IQR)	3149	62 (55–69)	62 (54–68)	60 (53–68)	63 (56–70)	< 0.001
Female	3149	924 (29)	138 (31)	251 (31)	535 (28)	0.3
Non-Caucasian	3149	415 (13)	59 (13)	106 (13)	250 (13)	> 0.9
T stage	3149					< 0.001
T1		332 (11)	12 (2.7)	42 (5.2)	278 (15)	
T2		330 (10)	22 (5.0)	68 (8.4)	240 (13)	
T3		1988 (63)	310 (70)	559 (69)	1119 (59)	
T4		499 (16)	99 (22)	143 (18)	257 (14)	
Grade	3149					< 0.001
G1–G2		633 (20)	28 (6.3)	145 (18)	460 (24)	
G3		1323 (42)	160 (36)	347 (43)	816 (43)	
G4		1193 (38)	255 (58)	320 (39)	618 (33)	
Median no. of removed nodes (IQR)	1255	4 (1–8)	4 (2–9)	4 (1–8)	–	0.3
Median no. of positive nodes (IQR)	1255	0 (0–1)	2 (1–3)	–	–	< 0.001
Systemic therapy received	3149	1601 (51)	236 (53)	380 (47)	985 (52)	0.025

^a^Kruskal-Wallis rank-sum test; Pearson’s chi-square test

The median number of removed nodes was four for both the pN1 (IQR, 2–9) and pN0 (IQR, 1–8) patients. The median number of positive nodes was two for the pN1 patients (IQR, 1–3). The rates of postoperative systemic therapy exposure were 53% for the pN1 patients, 47% for the pN0 patients, and 52% for the pNx patients.

### Cancer-Specific Mortality, Overall Mortality, and Cox Regression Analyses

**Fig. 1:**
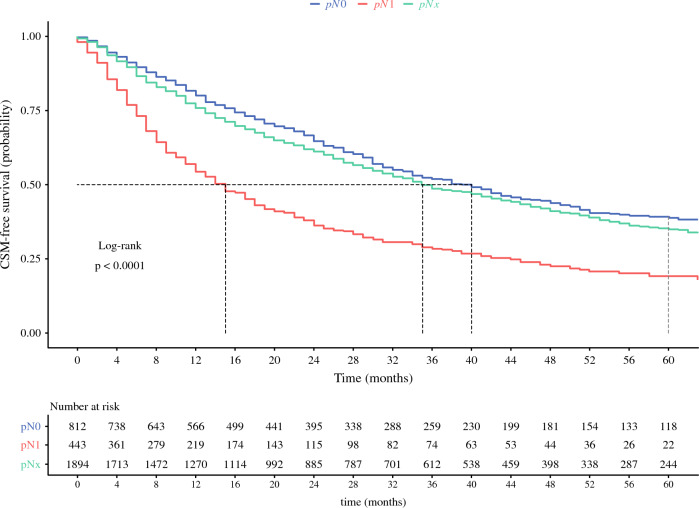
Kaplan-Meier curves depicting five-year cancer-specific mortality (CSM)-free survival according to pathological N-stage in 3149 metastatic renal cell carcinoma (mRCC) patients within the Surveillance, Epidemiology, and End Results (SEER) database (2010–2018)

The median CSM-free survival for the overall cohort was 33 months. According to pathologic lymph node stage, the median CSM-free survival was 15 versus 40 versus 35 months for pN1, pN0 and pNx, respectively (*P* < 0.001, log-rank test; Fig. 1). The 5-year CSM-free survival was 19% versus 39% versus 35% for pN1, pN0, and pNx, respectively. In univariable Cox regression models, the statistically significant predictors of CSM were pN1 stage (HR, 2.1; *P* < 0.01), T2 (HR, 1.3; *P* = 0.01), T3 (HR, 1.8; *P* < 0.01), T4 (HR, 2.0; *P* < 0.01), G3 (HR, 1.5; *P* < 0.01), G4 (HR, 2.5; *P* < 0.01), female sex (HR, 1.3; *P* < 0.1), year of diagnosis (HR, 1.0; *P* < 0.01), and systemic therapy exposure (HR, 1.3; *P* < 0.01) (Table 2). In multivariable Cox regression analyses, with adjustment for these variables, pN1 was an independent predictor of higher CSM (HR, 1.88; *P* < 0.01). Additionally, independent predictor status also was achieved by pNx (HR, 1.22; *P* < 0.01), T2 (HR, 1.34; *P* < 0.01), T3 (HR, 1.52; *P* < 0.01), T4 (HR, 1.63; *P* < 0.01), G3 (HR, 1.42; *P* < 0.01), G4 (HR, 2.17; *P* < 0.01), female sex (HR, 1.15; *P* = 0.01), and year of diagnosis (HR, 0.93, *P* < 0.01). The median overall survival according to pathologic lymph node stage was 14 versus 36 versus 32 months for pN1, pN0 and pNx, respectively (*P* < 0.001, log-rank test; Fig. S1). The 5-year overall survival rates were 16% versus 36% versus 31% for pN1, pN0, and pNx, respectively. In multivariable Cox regression analyses, pN1 was an independent predictor of higher OM (HR, 1.95; *P* < 0.001; Table S1).

**Table 2:** Cox regression analyses predicting cancer-specific mortality (CSM) for metastatic clear-cell renal cell carcinoma patients

	Univariable		Multivariable^a^	
	HR (95% CI)	*P* value	HR (95% CI)	*P* value
N stage (N0 ref.)				
N1	**2.10 (1.81–2.45)**	**<** **0.01**	**1.92 (1.63–2.26)**	**<** **0.01**
NX	1.12 (0.99–1.26)	0.06	**1.26 (1.10–1.43)**	**<** **0.01**
T stage (T1 ref.)				
T2	**1.34 (1.07–1.67)**	**0.01**	**1.41 (1.10–1.79)**	**<** **0.01**
T3	**1.79 (1.50–2.14)**	**<** **0.01**	**1.57 (1.29–1.92)**	**<** **0.01**
T4	**2.02 (1.65–2.47)**	**<** **0.01**	**1.71 (1.36–2.14)**	**<** **0.01**
Grade (G1–G2 ref.)				
G3	**1.49 (1.30–1.72)**	**<** **0.01**	**1.48 (1.26–1.73)**	**<** **0.01**
G4	**2.45 (2.12–2.82)**	**<** **0.01**	**2.31 (1.97–2.72)**	**<** **0.01**
Age	1.00 (0.99–1.01)	0.69	1.00 (0.99–1.01)	0.06
Female	**1.13 (1.03–1.26)**	**0.01**	**1.16 (1.04–1.28)**	**0.01**
Non-Caucasian	1.06 (0.92–1.22)	0.38	1.05 (0.95–1.26)	0.19
Year of diagnosis	**0.95 (0.93–0.97)**	**<** **0.01**	**0.93 (0.90–0.95)**	**<** **0.01**
Systemic therapy	**1.20 (1.08–1.34)**	**<** **0.01**	1.06 (0.88–1.19)	0.22

*P* < 0.05 values are given in bold

*HR* hazard ratio, *CI* confidence interval

^a^Covariates in the multivariable model: age at diagnosis, sex, year of diagnosis, N stage, T stage, grade, systemic therapy

### Subgroup Analyses Examining the Benefit of Postoperative Systemic Therapy for Patients With Pathologically Confirmed Lymph Node Invasion

The median CSM-free survival for pN1 mRCC patients who received postoperative systemic therapy was 15 versus 10 months for the patients who did not receive postoperative systemic therapy. After multivariable adjustment, postoperative systemic therapy status represented an independent predictor for lower CSM (HR, 0.73; *P* < 0.01) and OM (HR, 0.71; *P* < 0.01).

## Discussion

Historically, the presence of lymph node invasion represented an independent predictor of worse survival for cytoreductive nephrectomy (CN) mRCC patients.^2^ However, no contemporary data support the prognostic significance of pN1 for CN-treated mRCC patients. Consequently, we addressed this knowledge gap and investigated the prognostic significance of pathologic lymph node status in a contemporary cohort of surgically treated mRCC patients.

Our analyses resulted in several important observations. First, the population of CN-treated mRCC patients represents a relatively rare entity, in which patient counts generally are low. Consequently, population-based data are necessary for meaningful assessments of tumor characteristics. In the current study, we identified 3149 mRCC patients in the SEER database who underwent cytoreductive nephrectomy between 2010 and 2018. This sample size was comparable with those of other large-scale analyses. For example, in a previous SEER analysis, Zhang et al.^5^ identified 2352 CN mRCC patients between 2010 and 2015.

Second, according to the current analysis, 1255 CN mRCC patients (40%) underwent LND. Of those patients, 443 (35%) were labeled pN1 and 812 (65%) were labeled pN0. The rate of pN1 patients in the current study is similar to those reported in previous studies. Specifically, Feuerstein et al.^6^ reported a 33% pN1 rate in mRCC patients (*n* = 258). Similarly, Tappero et al.^7^ reported a 25% pN1 rate in surgically treated mRCC patients (*n* = 814) with primary tumors 4 cm in size or smaller. Notably, a comparison of pN1 and pN0 patients showed that the pN1 patients had higher rates of T4 tumors (22% pN1 vs 18% pN0; *P* = 0.04) and G4 tumors (58% pN1 vs 39% pN0; *P* < 0.01). Conversely, the rate of G1–G2 tumors was significantly lower among the pN1 patients (6.3% pN1 vs 18% pN0; *P* < 0.01).

Third, we tested for CSM differences in these patients according to pathologic N stage. Specifically, the 5-year CSM-free rates were 19% versus 39% versus 35% for pN1, pN0 and pNx, respectively (*P* < 0.01). In multivariable analyses, pN1 was an independent predictor of higher CSM (HR, 1.88; *P* < 0.01).

To the best of our knowledge, we are the first to confirm the prognostic significance of pathologic lymph node invasion in contemporary, surgically treated mRCC patients. Consequently, our results cannot be compared directly with similar, contemporary studies that relied on immunotherapy-era patients. However, previous historical studies demonstrated the same phenomenon. These studies applied to both pre-TKI therapy-era patients and TKI-era patients.^2,3^ In these studies, pN1 stage represented an independent predictor of worse survival in the same fashion as was recorded in the current study.

To further test the prognostic significance of pathologic lymph node invasion, we reapplied the methodology described earlier, relying on overall mortality as the end point. After multivariable adjustment, pN1 remained an independent predictor of higher OM (HR, 1.95; *P* < 0.01), further adding to the robustness of our data.

Taken together, our observations indicate that even in this immunotherapy era, pN1 stage harbors added value regarding prognostic stratification of CN mRCC patients. Specifically, when LND is performed, those with pN1 status exhibit significantly worse survival than those with pN0 status. Such stratification may improve clinical decision-making regarding early versus delayed use of systemic therapy for mRCC patients who underwent CN.

Finally, we tested the benefit of postoperative systemic therapy (ST) administration for pN1 patients based on the hypothesis that early ST administration, captured in the current database, may be associated with a stronger protective effect when CSM represented an end point. Our analyses indeed recorded lower CSM rates for the patients treated with postoperative ST than for those who were not (HR, 0.73; *P* < 0.01). This observation further validates that pathologic N1 status may indeed improve decision-making regarding postoperative ST administration. It may be argued, that patients with confirmed pN1 status should ideally receive the earliest postoperative ST.

Despite the novelty of our findings, several limitations of our study need to be acknowledged. First, our data reflect CSM patterns of North American CN mRCC patients. Consequently, estimates shown in this report cannot be applied to CN mRCC patients outside the United States.

Second, the SEER database does not allow stratification or adjustment of the analyses according to Memorial Sloan Kettering Cancer Center (MSKCC) or International mRCC Database Consortium (IMDC) criteria. This limitation is shared with all previous SEER and National Cancer Database (NCDB) analyses.

Third, the SEER database does not provide detailed information about the composition or the exact timing of postoperative systemic therapy (immediate vs deferred). Moreover, our study included only patients with mRCC diagnosed between 2010 and 2018, a period in which immunotherapy was recommended as a first-line systemic treatment option. However, it is important to note that not all patients who received systemic therapy during this period necessarily received immunotherapy. Finally, our report represents a retrospective analysis with high potential for selection biases.

## Conclusions

Pathologically confirmed lymph node invasion independently predicted higher CSM and OM in surgically treated mRCC patients. For pN1 mRCC patients, use of postoperative systemic therapy was associated with lower CSM and OM. Consequently, N stage should be considered for both individual patient counseling and clinical decision-making.

### Supplementary Information

Below is the link to the electronic supplementary material.

Supplementary file1 (DOCX 3452 kb)

## Data Availability

All data generated for this analysis were from the Surveillance, Epidemiology, and End Results Research Plus (SEER) database. The code for the analyses will be made available upon request.

## References

[1] Marchioni M, Amparore D, Magli IA (2022). Potential benefit of lymph node dissection during radical nephrectomy for kidney cancer: a review and critical analysis of current literature. Asian J Urol..

[2] Lughezzani G, Capitanio U, Jeldres C (2009). Prognostic significance of lymph node invasion in patients with metastatic renal cell carcinoma: a population-based perspective. Cancer..

[3] Trinh QD, Sukumar S, Schmitges J (2013). Effect of nodal metastases on cancer-specific mortality after cytoreductive nephrectomy. Ann Surg Oncol..

[4] R: The R Project for Statistical Computing. Retrieved 4 June 2023 at https://www.r-project.org/.

[5] Zhang Y, Hu J, Yang J (2022). Selection of optimal candidates for cytoreductive nephrectomy in patients with metastatic clear cell renal cell carcinoma: a predictive model based on SEER database. Front Oncol..

[6] Feuerstein MA, Kent M, Bernstein M, Russo P (2014). Lymph node dissection during cytoreductive nephrectomy: a retrospective analysis. Int J Urol..

[7] Tappero S, Barletta F, Piccinelli ML (2023). The association between cytoreductive nephrectomy and overall survival in metastatic renal cell carcinoma with primary tumor size ≤ 4 cm. Eur Urol Focus..

