# Supplementary material for: Prognostic Significance of Pathologic Lymph Node Invasion in Metastatic Renal Cell Carcinoma in the Immunotherapy Era
Source: Ann Surg Oncol. 2023 Oct 10;30(13):8780–5. doi: 10.1245/s10434-023-14367-6 (PMC10625944; doi:10.1245/s10434-023-14367-6)
Supplement: Supplementary file 1 — Supplementary file1 (DOCX 3452 kb) [file 10434_2023_14367_MOESM1_ESM.docx]

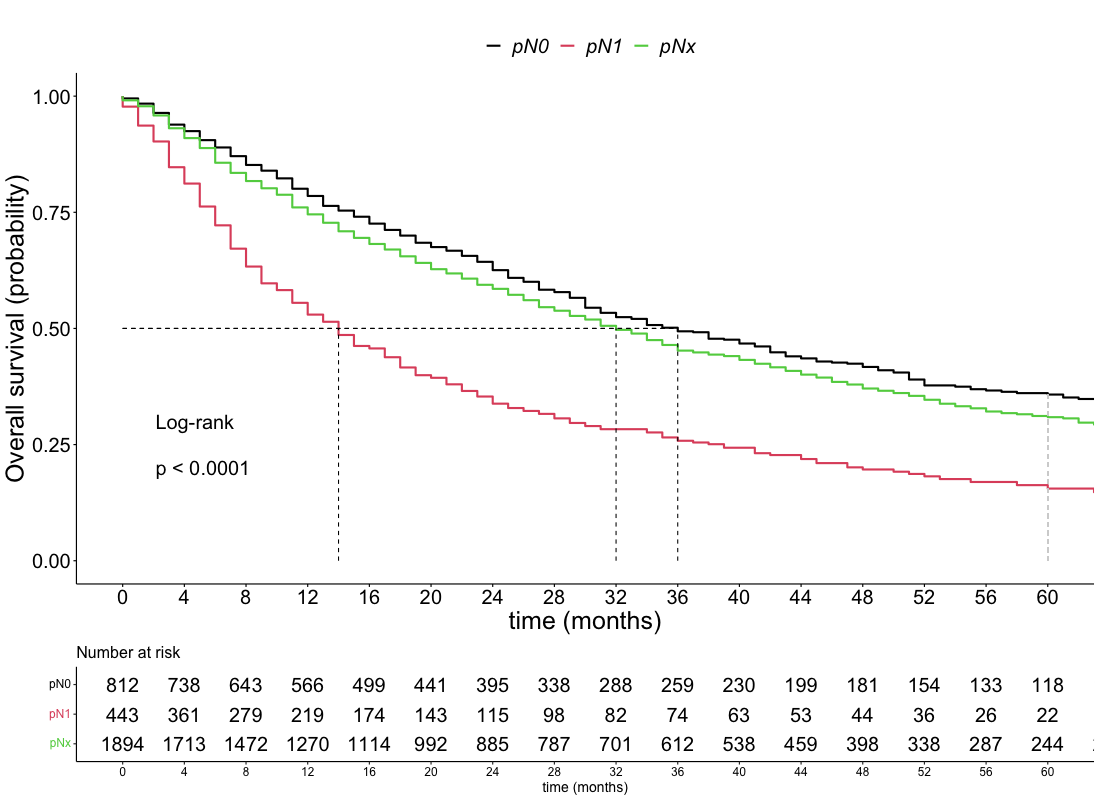


**Supp.Figure:** Kaplan-Meier curves depicting five-year overall survival (OS) according to pathological N-stage in 3,149 metastatic renal cell carcinoma (mRCC) patients within the Surveillance, Epidemiology, and End Results (SEER) database (2010-2018).

| **Supp.Table:** Cox regression analyses predicting overall mortality (OM) for metastatic clear-cell renal cell carcinoma patients | | | | | | | | |
| --- | --- | --- | --- | --- | --- | --- | --- | --- |
|  | **Univariable** | |  | | **Multivariable*** | |  | |
|  | HR | | p-value | | HR | | p-value | |
| **N-stage (N0 Ref.)** |  |  | |  | |  | |  |
| N1 | **2.08 (1.80-2.41)** | **<0.01** | | **1.95 (1.65-2.27)** | | **<0.01** | |  |
| NX | 1.12 (1.01-1.26) | **0.03** | | **1.26 (1.11 -1.42)** | | **<0.01** | |  |
|  |  |  | |  | |  | |  |
| **T-stage (T1 Ref.)** |  |  | |  | |  | |  |
| T2 | **1.24 (1.01-1.53)** | **0.03** | | **1.28 (1.02-1.60)** | | **0.02** | |  |
| T3 | **1.63 (1.38-1.93)** | **<0.01** | | **1.47 (1.23-1.76)** | | **<0.01** | |  |
| T4 | **1.84 (1.52-2.22)** | **<0.01** | | **1.60 (1.30-1.97)** | | **<0.01** | |  |
|  |  |  | |  | |  | |  |
| **Grade (G1-G2 Ref.)** |  |  | |  | |  | |  |
| G3 | **1.40 (1.23-1.60)** | **<0.01** | | **1.39 (1.20-1.60)** | | **<0.01** | |  |
| G4 | **2.16 (1.89-2.46)** | **<0.01** | | **2.07 (1.77-2.40)** | | **<0.01** | |  |
|  |  |  | |  | |  | |  |
| Age | 1.00 (0.99-1.01) | 0.69 | | 1.01 (1.01-1.02) | | **0.01** | |  |
| Female | **1.15 (1.04-1.27)** | **<0.01** | | **1.17 (1.06-1.30)** | | **0.01** | |  |
| Non-Caucasian | 1.07 (0.92-1.22) | 0.38 | | 1.06 (0.92-1.22) | | 0.19 | |  |
| Year of diagnosis | **0.96 (0.94-0.98)** | **<0.01** | | **0.94 (0.92-0.97)** | | **<0.01** | |  |
| Systemic therapy | **1.14 (1.04-1.26)** | **<0.01** | | 1.02 (0.93-1.13) | | 0.57 | |  |
| *Covariates in multivariable model: age at diagnosis, sex, year of diagnosis, N-stage, T-stage, grade, ethnicity, systemic therapy | | | | | | | | |
